# Prediction of the bacterial shape in urinary tract infections with the Sysmex UF-1000i analyser: technical note

**DOI:** 10.1097/MS9.0000000000000701

**Published:** 2023-07-27

**Authors:** Noussaiba Benhamza, Adnane Aarab, Soumya Farih, Abderrazak Saddari, Loubna Yacoubi, Elmostapha Benaissa, Yassine Ben Lahlou, Mostafa Elouennass, Adil Maleb

**Affiliations:** a Laboratory of Microbiology, Mohamed VI Teaching Hospital; b Research team ‘Cell Biology and Pharmacology Applied to Health Sciences’, Faculty of Medicine and Pharmacy, Mohammed First University, Oujda; c Department of Bacteriology, Mohammed V Teaching Military Hospital; d Epidemiology and Bacterial Resistance Research Team/BIO-INOVA Centre, Faculty of Medicine and Pharmacy, University Mohammed V, Rabat, Morocco

**Keywords:** morphology discrimination UTI, urinary tract infections, UF-1000i, B_FSC

## Abstract

**Background:**

The aim of our study was to explore the utility of the Sysmex UF-1000i analyzer as a rapid screening tool for urinary tract infections (UTI) and its ability to predict bacterial shape in order to help physicians choose the appropriate empiric treatment.

**Materials and methods:**

This is a retrospective study, including 1023 urine cytobacteriological examinations. Urines were processed according to the recommendations of the medical microbiology reference system (REMIC). Using the Sysmex Uf-1000i analyzer, the authors evaluated bacteria forward scatter (B_FSC) and fluorescent light scatter (B_FLH) in a preliminary discrimination step for UTI caused by bacilli or cocci bacteria.

**Results:**

The authors got 1023 positive samples. Comparing baccili and cocci bacteria, the authors observed a statistically significant difference for B_FSC but not for B_FLH. The values of B_FLH are very close for the four categories of microorganisms compared (bacilli, cocci, bacilli–cocci association, and yeasts). For these same categories, tests show different values for the B_FSC. A separate analysis of the B_FSC values for bacilli shows that their distribution is relatively homogeneous and exhibits a peak between 20 and 30 ch.

**Conclusion:**

Dimensional parameters of bacteria generated by UF-1000i could be a rapid and useful tool for predicting the bacterial shape causing UTI.

## Background

Highlights

- Using the Sysmex Uf-1000i analyzer, we evaluated bacteria forward scatter (B_FSC) and fluorescent light scatter (B_FLH) in a preliminary discrimination step for urinary tract infection caused by bacilli or cocci bacteria.
- Tests show different values for the B_FSC. A separate analysis of the B_FSC values for bacilli shows that their distribution is relatively homogeneous and exhibits a peak between 20 and 30 ch.
- Dimensional parameters of bacteria generated by UF-1000i could be a rapid and useful tool for predicting the bacterial shape causing urinary tract infection.

Urinary tract infection (UTI) is considered the most important bacterial infection in the general population^1–3^. It is the first healthcare-related infection and the second community infection^4^. The annual incidence of UTI worldwide is estimated at 175 million episodes^5^. The microbiological diagnosis of ITU is based on cytobacteriological examination of the urine (CBEU), which is the most prescribed test in medical microbiology^6^. Problems remaining in the results of CBEU are the low prevalence of positive samples, especially by contamination of samples, and the turnaround time for CBEU results is no less than 48 h. We cannot wait for 2 days before starting antimicrobial treatment in a symptomatic patient. So physicians usually start empiric antimicrobial therapy based on host characteristics and the ecology of the department. Unfortunately, the most active agents against Gram-negative bacteria are not very effective against Gram-positive bacteria^3,7^. An initial idea of the Gram characteristic of the bacteria involved in suspected UTI would certainly enhance the efficacy of empirical therapy^8,9^. Automated method overcomes these drawbacks by providing rapid results that could guide therapeutic management^7,10,11^. Several studies find that the evaluation of dimensional parameters of bacteria, derived from the distribution histograms in the bacterial channel [Bacteria Forward Scatter (B_FSC) and Bacteria Fluorescent Light Scatter (B_FLH)] of the modern cytometers can be useful in an extremely rapid etiological differentiation^7,12^.

The aim of our study was to evaluate the utility of the Sysmex UF-1000i analyzer as a rapid screening tool for UTI and its ability to predict bacterial shape (cocci or bacilli) in order to help physicians choose appropriate empiric treatment.

## Materials and methods

This is a retrospective study from 01 January 2020 to 30 June 2020, including 1023 urine cytobacteriological examinations (CBEU) with positive cultures. The CBEU were carried out in accordance with the standard in medical microbiology (REMIC)^4^. Urine cytology was performed on the Sysmex UF-1000i machine. Cultures, which represent the gold standard method for the diagnosis of UTI, were performed on Brillance UTI Agar Oxoid chromogenic media, and confirmation of the biochemical identification of bacteria was performed on the BD Phoenix 100 analyzer.

The Sysmex UF-1000i (Sysmex Co.) is a fluorescence flow cytometer designed for urine analysis. This technique provides analytical data for microbiological diagnosis. The cytometer ensures the detection and counting of bacteria in a specific analysis channel using a specific reagent device. The system is equipped with specific technical parameters that provide information on the size and staining characteristics of the particles identified and counted in this channel. The UF-1000i flow cytometer was used to analyze the urine samples according to the vendor’s protocol. This system allows the determination of particle size and uses specific fluorescent dyes (phenanthridine for nucleic acid staining and carbocyanine for membrane staining) for their counting and classification. This system allows recording fluorescence values for the bacterial channel, which are measured in arbitrary fluorescence units.

Our study consisted of exploring the correlation between the results of the culture (bacilli, cocci and/or yeasts) and the flow cryometry parameters provided by the Sysmex UF-1000i automaton. These parameters were B_FSC (Bacteria Forward Scatter) and B_FLH (Bacteria Fluorescent Light Scatter). They were expressed in arbitrary units (ch) and provided information about the examined microorganisms’ morphology. We have grouped the results into four categories (bacilli, cocci, bacilli–cocci association, and yeasts), then we established the distribution of B_FSC and B_FLH values in each category by using the Kruskal–Wallis rank sum test. In the B_FSC value, we were interested in checking the shape of the curve resulting from the distribution of values for bacilli, cocci, and their subpopulations (staphylococci and enterococci) using Microsoft Excel.

## Results

**Figure 1:**
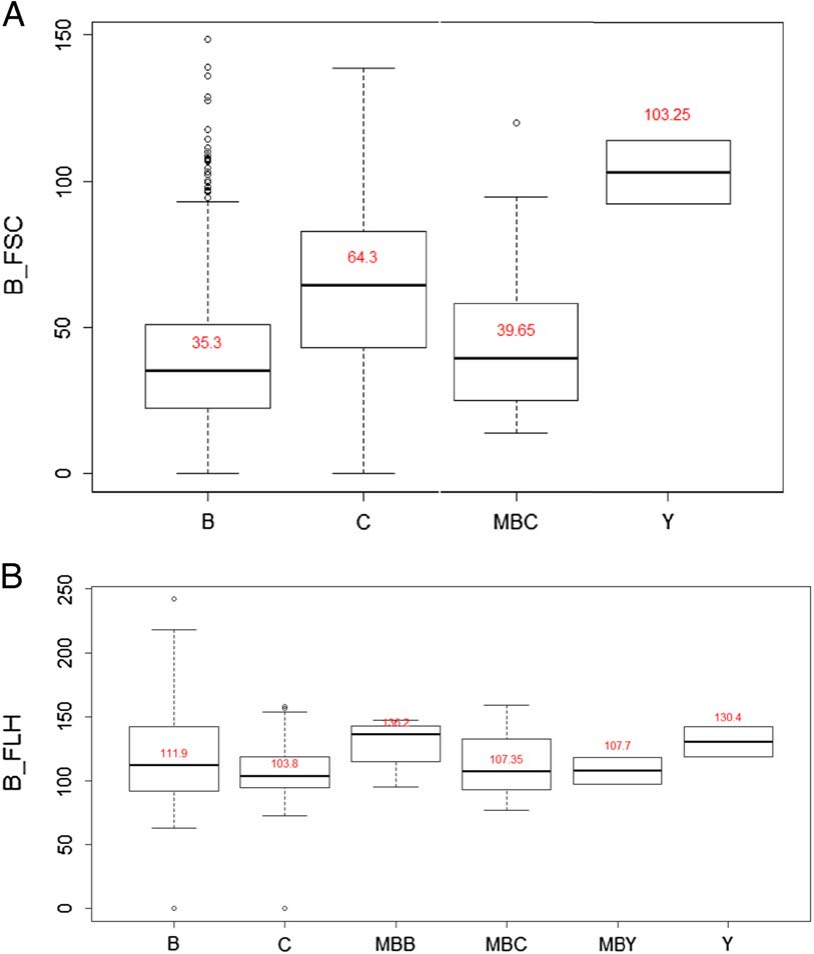
Distribution of B_FSC (A) and B_FLH (B) values (Kruskal–Wallis rank sum test). Boxes represent quartile ranges of B_FSC. The horizontal line in the middle of the boxes represents the median value. *P*-value=0.1001. B_FSC: Bacteria Forward Scatter, B_FLH: Bacteria Fluorescent Light Intensity, B: bacilli, C: Cocci, MBB: Bacilli and Cocci association, Y: Yeast.

**Figure 2:**
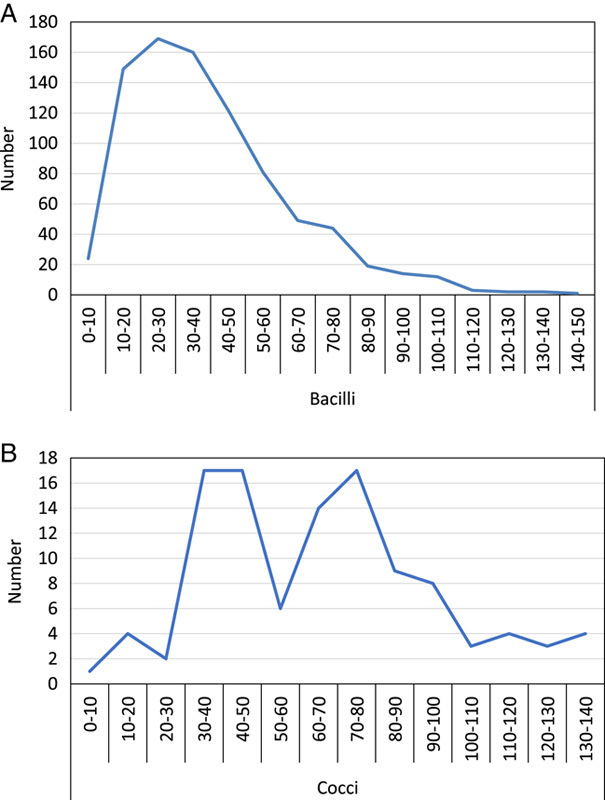
Distribution of B_FSC values for bacilli alone (*n*=851) (A) and for cocci alone (*n*=109) (B).

**Figure 3:**
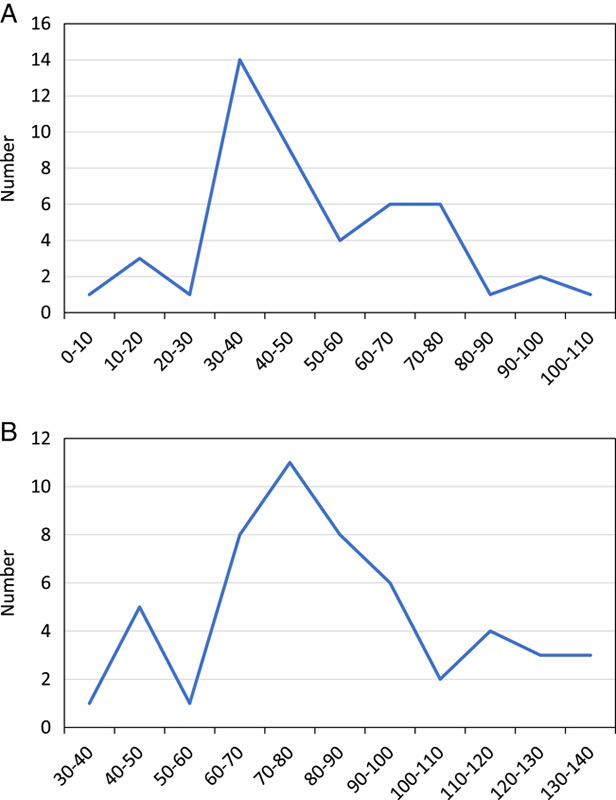
Distribution of B_FSC values for enterococci alone (A) and staphylococci alone (B) (*n*=109).

During the study period, we collected 1023 bacteria, of which 86.90% (*n*=889) were bacilli (Table 1). Table 2 shows the number of germs isolated alone or in association with other germs. The Kruskal–Wallis rank sum test shows the distribution values of B_FLH (Fig. 1A) and B_FSC (Fig. 1B). This test shows that values of B_FLH are very close in the four categories of microorganisms compared. For these same categories, the Kruskal–Wallis rank sum test shows different values for the B_FSC. A separate analysis of the B_FSC values for the bacilli shows that their distribution is relatively homogeneous and exhibits a peak between 20 and 30 ch consistent with the results of the Kruskal–Wallis rank sum test (Fig. 2A). However, cocci’s distribution values of B_FLH were heterogeneous with a first peak between 30 and 50 ch, and a second peak between 70 and 80 ch (Fig. 2B). These two peaks correspond to enterococci (Fig. 3A) and staphylococci (Fig. 3B), respectively.

**Table 1:** Isolated strains from positive urine samples (*n*=1023).

Class	Genus	Species	*n* (%)
Bacilli (*n*=889; 86.90%)	*Escherichia*	*coli*	627 (61.29)
	*Klebsiella*	*pneumoniae*	148 (14.47)
		*oxytoca*	10 (0.98)
	*Pseudomonas*	*aeruginosa*	34 (3.32)
	*Enterobacter*	*cloacae*	28 (2.74)
		*aerogenes*	4 (0.39)
	*Proteus*	*mirabilis*	10 (0.98)
	*Acinetobacter*	*baumannii*	10 (0.98)
	*Citrobacter*	*koseri*	3 (0.29)
		*farmeri*	1 (0.10)
		*braakii*	1 (0.10)
	*Morganella*	*morganii*	3 (0.29)
		*Autres*	10 (0,98)
Cocci (*n*=130; 12.71%)	*Enterococcus*	*faecalis*	54 (5.28)
		*faecium*	12 (1.17)
	*Staphylococcus*	*epidermidis*	21 (2.05)
		*aureus*	15 (1.47)
		*haemolyticus*	11 (1.08)
		*saprophyticus*	3 (0.29)
		*hominis*	2 (0.20)
		*capitis*	1 (0.10)
		*simulans*	1 (0.10)
		*Autres*	10 (0.98)
Yeasts (*n*=4; 0.39%)	*Candida*	*kefyr*	1 (0.10)
		*parapsilosis*	1 (0.10)
		*albicans*	1 (0.10)
	*Saccharomyces*	*cerevisiae*	1 (0.10)

**Table 2:** Germs isolated individually or in association (*n*=986).

Isolat	*n* (%)
Bacilli alone	851 (86.31)
Cocci alone	109 (11.05)
Yeast alone	2 (0.20)
Bacilli + Bacilli association	4 (0.41)
Bacilli + Cocci association	18 (1.83)
Bacilli + Yeast association	1 (0.10)
Cocci + Yeast association	1(0.10)

## Discussion

UTIs are common conditions that require prompt diagnosis and treatment^13^. Culture requires at least 24 h of incubation and an additional 24 h for the identification of urine germs^4^. In addition, CBEU is expensive and often yields negative results^13^. This affects physician’s decision-making, patient’s treatment, and laboratory workload^3,7^. Among many cytobacteriological examinations performed in the microbiology laboratory, CBEU is one of the analyzes where microscopic examination after Gram stain is not systematic^4^. In fact, Gram staining of centrifuged urine has been used in some microbiology laboratories as a preculture screening method, but for this purpose, despite its high sensitivity and specificity of 90% compared to culture, it is not very effective because it represents an additional workload when it is necessary to examine large series of urine^14^. The solution to this constraint would be the use of a reliable and automated screening test. The Sysmex UF-1000i is a second-generation automated urinary analyzer, which uses fluorocytometry in flow and a red semiconductor laser to identify and quantify urine elements present in the sample^7,15^. Sysmex UF-1000i analyzer has an analysis channel dedicated to the precise counting of bacteria^3,16^. In this channel, size and nucleic acid content of bacteria are assessed using two parameters, B_FSC and B_FLH expressed in arbitrary units (ch), which provide information about the morphology of microorganisms^3,16^.

A statistical analysis of our results finds that bacilli and cocci have similar B_FLH values. Both have values between 80 and 120 ch. The values of B_FSC of bacilli and those of cocci are different. For bacilli, these values are between 20 and 30 ch. For cocci, the first values are between 30 and 50 ch, while the second values are between 70 and 80 ch. The first peak corresponds to enterococci and the second corresponds to staphylococci, respectively.

The explanation for the difference in peaks between the two Gram-positive cocci would be their different grouping modes^11^. Indeed, several studies have shown that enterococci can have bacterial distribution profiles similar to those of Gram-negative bacteria, due to the formation of short chains^11^. The differences between cocci and bacilli may be explained by the fact that cocci aggregate in clusters or form chains, resulting in a larger and more variable size cocci aggregates compared to Gram-negative bacilli, which tend to stay in suspension as single cells^7^. These results in higher and wider distribution B-FSC values for cocci compared to bacilli, which exhibit lower and more homogeneous B-FSC values^7^. In a study conducted by Jefferson and Mendoza^3^, the median B_FSC was 20.05 ch in Gram-negative and 48.1 ch in Gram-positive (*P*<0.0001). Out of 294 samples with a B_FSC value <30 ch; 256 (87.4%) were Gram-negative, 7 (2.4%) Gram-positive, and 31 (10.5%) mixed growth. Two hundred fifty samples had a B_FSC value <25 ch; 224 (89.6%) were Gram-negative, 2 (0.8%) Gram-positive, and 24 (9.6%) mixed growths^3^. B_FSC showed a higher specificity at a cutoff of 25 ch (87.7%) than 30 ch (80,8%), making it easier to obtain information on the causative agent in a few minutes and therefore optimize the management of suspicious UTI and guide antibiotic treatment^3^. In practice, UTI are caused in most cases by Gram-negative bacilli such as *Enterobacteriaceae* (*E. coli*, etc.), then nonfermenting Gram-negative bacilli (*Pseudomonas* spp., etc.) and Gram-cocci positive (*Enterococcus* spp., *Streptococcus* spp., and *Staphylococcus* spp.)^6^. The UF-1000i analyzer detects this morphological difference which can be available in a few minutes and be fully automated without additional equipment^2,4,7,17–21^.

Our study had some limitations: we were unable to test samples with ‘false positive’ results generated by Sysmex UF-1000i and results from polymicrobial and contaminated cultures. We did not analyze the possible antibacterial treatments, which can be administered to patients before the microbiological examination so that the shape of the bacteria can be influenced by the antibiotic therapy, which could influence the results or the diagrams of typical dispersion.

## Conclusion

Our study finds that the analysis of the dimensional parameters of bacteria generated by UF-1000i could be a useful tool for the rapid detection of UTI, by predicting the bacterial shape (cocci or bacillus). This method appears efficient in accelerating the time limit of results, especially in emergencies.

## Ethical approval and consent to participate

The study was carried out in accordance with the Helsinki Declaration. The study was conducted on anonymous biological samples. It does not concern any personal data that could directly or indirectly identify a specific person.

## Consent for publication

Not applicable.

## Sources of funding

No sources of funding were obtained for this project.

## Author contribution

All authors have made substantial contributions to all of the following: the conception and design of the study, drafting the article and revising it critically for important intellectual content, final approval of the version to be submitted.

## Conflicts of interest disclosure

The authors declare that they have no conflicts of interest.

## Data availability statement

All the data and material were presented in the main paper. The datasets used and/or analyzed during the current study are available from the corresponding author upon reasonable request.

## Provenance and peer review

Not commissioned, externally peer reviewed.
